# Corrigendum to: Posters

**DOI:** 10.1002/2211-5463.12764

**Published:** 2019-12-02

There are corrections to three poster abstracts in the supplement for the 44th FEBS Congress, From Molecules to Living Systems, Krakow, Poland, July 6–11, 2019 1.

The full author list for the poster **P‐35‐147** is as follows:

**P‐35‐147**

**Modulation of breast cancer cells growth rate via the inhibitory effect of different chemical substances**

**Harun M. Said** ^**1**^ **, Güler Gül** ^**1**^ **, Gül Güner Akdoğan** ^**3**^ **, Yasemin Soysal** ^**2**^

^1^ *Department of Molecular Medicine, Higher Institute of Health Sciences, Dokuz Eylül University, Izmir, Turkey*, ^2^ *Department of Molecular Medicine (MOT), Graduate School of Health Sciences, Dokuz Eylül University, Izmir, Turkey*, ^3^ *Department of Biochemistry, School of Medicine, Izmir University of Economics, Izmir, Turkey*

The poster **P‐01‐059** was omitted in error and is reproduced here:

**P‐01‐059**

**The effect of quercetin on oxidative stress parameters in the fructose‐induced experimental metabolic syndrome model**

D. Özer*, K. Kocaman*, B. Narli*, C. Yilmaz Demirtaş*

*Gazi University Medical Faculty, Department of Medical Biochemistry, Ankara, Turkey*

The aim of this study is to investigate the possible protective and therapeutic effects of the administration of quercetin in the liver tissue on metabolic syndrome components in the rat fructose‐mediated metabolic syndrome model. In our study, the effect of quercetin on malondialdehyde (MDA), advanced oxidation protein products (AOPP), and nitric oxide (NO) levels of liver tissue was investigated. Twenty‐four Sprague‐Dawley rats were divided into 4 groups (*n* = 6): control, fructose, quercetin, and fructose+quercetin. In a 10‐week trial period, quercetin was administered via oral gavage at a dose of 15 mg/kg daily, and fructose was administered in drinking water at 20%. At the end of the 10th week, the animals were sacrificed under anesthesia. Then, blood and liver tissue samples were taken; serum glucose, lipid profile, and insulin levels were measured; and insulin resistance was calculated. MDA, AOPP, and NO levels were determined in the liver tissue of rats, and by using the SPSS program, the results were compared with the treated groups and control groups. MetS was successfully formed; hypertension, hyperlipidemia, hyperglycemia, and insulin resistance were detected in the fructose groups. According to the control group, there was a statistically significant increase in MDA and AOPP levels in the fructose groups, but the increase in NO levels was not significant. When quercetin was administered with fructose, MDA levels were found to be significantly higher than in the control group, but no difference was observed when compared to the fructose group. When quercetin was given to the fructose group, AOPP levels were decreased significantly compared to the fructose group, and the decrease in NO levels was not significant. According to these results, we believe that the use of MetS models in comprehensive projects on the targets and mechanisms of action of quercetin, which is supported by transcriptomic, proteomic, and metabolomic studies, will shed light on the pathogenesis and treatment of MetS.

*The authors marked with an asterisk contributed equally to the work.

The abstract for poster P‐37‐014 (“Identification and analysis of super‐enhancers as novel biomarkers and potential therapeutic targets for age‐associated diseases”) stated that the research was supported by Russian Foundation for Basic Research (grant 16‐33‐60228). This grant number is incorrect. The correct statement is as follows: The research was supported by Russian Foundation for Basic Research (grant 19‐34‐80052).
